# Understanding the role of risk preferences and perceptions in vaccination decisions and post-vaccination behaviors among U.S. households

**DOI:** 10.1038/s41598-024-52408-6

**Published:** 2024-02-07

**Authors:** Jianhui Liu, Bachir Kassas, John Lai, Jaclyn Kropp, Zhifeng Gao

**Affiliations:** 1 Food and Resource Economics Department, Institute of Food and Agricultural Sciences, University of Florida, 2120 McCarty B, Gainesville, FL 32611 USA; 2 Food and Resource Economics Department, Institute of Food and Agricultural Sciences, University of Florida, 1099 McCarty B, Gainesville, FL 32611 USA; 3 Food and Resource Economics Department, Institute of Food and Agricultural Sciences, University of Florida, 1109 McCarty B, Gainesville, FL 32611 USA; 4 Food and Resource Economics Department, Institute of Food and Agricultural Sciences, University of Florida, 1157 McCarty B, Gainesville, FL 32611 USA; 5 Food and Resource Economics Department, Institute of Food and Agricultural Sciences, University of Florida, 1155 McCarty A, Gainesville, FL 32611 USA

**Keywords:** Health care, Risk factors

## Abstract

COVID-19 vaccines play a critical role in protecting against infection and transmission of the virus. Therefore, understanding public perceptions of COVID-19 vaccines is essential for successful vaccine promotion. Previous literature reported strong associations between vaccination decisions and several sociodemographic variables. However, knowledge about how behavioral factors, including risk perceptions and preferences, impact individuals’ attitudes towards receiving COVID-19 vaccination is currently lacking. Using data from a nationally representative survey of 1050 US adults, this study investigates the correlation between individuals’ decisions to receive COVID-19 vaccination and both their risk perceptions and preferences. Additionally, we investigate post-vaccination behavior by measuring individuals’ participation in three different groups of activities that vary by their degree of social exposure. We find strong correlations between vaccination decisions and four measures of risk preference and risk perception. We also find associations between the four risk measures and individuals’ behaviors post-vaccination. We shed light on the main factors discouraging the uptake of COVID-19 vaccines, as well as public opinions regarding the performance of different organizations in addressing the COVID-19 pandemic, and grocery store policies to prevent COVID-19 infections. Our study provides critical information that can help policymakers communicate more effectively with the public and promote vaccine uptake among population groups and geographic areas with higher anti-vaccine sentiments.

## Introduction

Following the World Health Organization’s (WHO) declaration of COVID-19 as a global pandemic^1^, worldwide efforts were mobilized to limit the transmission and public health impacts of COVID-19. Initial prevention measures, including lockdowns and other mitigation behaviors (e.g., social distancing, face masks, and sanitation), succeeded in slowing the transmission of the virus^2^. However, the sustainability of these methods is not pragmatic due to adverse effects on the economy and global supply chains. More enduring solutions that have emerged included the use of COVID-19 vaccines. In the United States (US), the Food and Drug Administration (FDA) issued emergency use authorization for two COVID-19 vaccines, Pfizer-BioNTech and Moderna, in December 2020. This was followed by a third vaccine by Johnson and Johnson, which was approved in February 2021. Extensive research on these vaccines showed decreased transmission rates, along with reduced severity of COVID-19 symptoms^3^.

Vaccination treatments have been deployed in human populations for centuries to prevent the spread of various transmissible diseases, such as Influenza, tuberculosis, and pertussis^4^. However, compared to regular seasonal Influenza, and other previous global pandemics, such as the 2009 H1N1 Influenza, COVID-19 carries more severe symptoms and much higher infection and mortality rates^5^. COVID-19 vaccines have proven to be crucial tools for protecting public health^6^. Despite the effectiveness of COVID-19 vaccines in preventing severe symptoms, many people remain unvaccinated^7^. Thus, it is important to promote the uptake of COVID-19 vaccines among US households, especially during periods of high transmissibility and infection rates^3^.

Immunization programs are not considered successful until high crowd acceptance and high inoculation rates are achieved^8^, leading to what is commonly referred to as “herd immunity”. Reaching herd immunity depends on the decisions and behaviors of the population. As more people get vaccinated, the potential for reaching herd immunity increases, and mutated virulent strains or variants may become less likely to emerge^9^. Furthermore, achieving herd immunity could be expedited by increasing the pace of vaccination and reducing vaccine hesitancy^10^. For this reason, it is critical to understand public perceptions and acceptance of COVID-19 vaccination. This knowledge is extremely helpful in informing the development of effective strategies to encourage higher vaccine acceptance and uptake. In this study, we investigate behavioral characteristics influencing COVID-19 vaccination decisions. Specifically, we focus on how individuals’ risk perceptions and risk preferences correlate with their attitudes toward COVID-19 vaccination. We also analyze the main factors discouraging COVID-19 vaccination and examine changes in behavior post-vaccination.

Vaccine hesitancy can be defined as voluntary refusal or delay in accepting available vaccine, and it is driven by many context-specific factors that vary across location, time, and type of vaccine^11,12^. During the development period of COVID-19 vaccines, several researchers found that requests for such vaccines were strongly correlated with multiple demographic factors, such as gender, race, education, political affiliation, and household characteristics^13–16^. Conversely, some individuals rejected vaccinations for reasons that included concerns about side effects, general distrust in all vaccines, and over-confidence about their own health conditions^17–20^. We contribute to this literature by examining how individuals’ risk perceptions and preferences correlate with their vaccination decisions, and by investigating vaccination plans for individuals who have not yet received the COVID-19 vaccine.

An individual’s risk perception can be defined as an intuitive assessment of losses and/or hazards relating to a specific action or situation^21^. This judgment process comprises the individual’s perception of the likelihood of a hazardous event and how the individual labels a situation in terms of the risk level (e.g., low/high risk)^22^. On the other hand, risk preferences define people’s consistent tendency or inclination toward risk in their decision-making process^23^. Risk perceptions and preferences are both important determinants of individual behavior under risk^24^. Thus, in this paper, we use four different risk measures to examine how risk perceptions and risk preferences correlate with COVID-19 vaccination decisions.

Our study presents several important contributions. First, it builds on existing literature by examining sociodemographic and behavioral factors influencing COVID-19 vaccination decisions in the US. Existing studies showed significant correlations between vaccination decisions and several sociodemographic factors, including race, gender, and employment status^8,16^. Behavioral factors, such as online misinformation, false claims, and conspiracy theories about the COVID-19 vaccine, were also shown to significantly influence the acceptance of vaccination^25,26^. Another study found that in Portugal, delays in COVID-19 vaccination, and refusal of vaccination, were highly associated with people’s age, income loss due to the pandemic, and intention to take the flu shot^27^. However, little is known about the extent to which risk perceptions and risk preferences influence individuals’ choices to receive the COVID-19 vaccine, although these risk measures were shown to be highly correlated with other health-related behaviors^23,28,29^. Our study highlights how several factors, including risk perceptions and risk preferences, lead to divergent COVID-19 vaccine sentiments among individuals in the US.

Second, this study contributes to the limited literature investigating changes in people’s daily activities post-vaccination. While Gaube et al.^57^ showed evidence that behaviors specific to health and safety are highly associated with risk perceptions, the effects on other regular daily activities are still ambiguous. Several studies showed that participation in physical activities, and other health-promoting behaviors, have significantly changed as a result of the pandemic^30–33^. However, little is known about how participation, or anticipated participation, in other daily activities, such as dining out and recreational behaviors, changed during the pandemic, and how COVID-19 vaccination influences participation in these activities. Our study adds to this literature by investigating these correlations.

Third, our study contributes to the growing literature on public attitudes related to food retail store policies during the COVID-19 pandemic, which are important considerations for a more comprehensive understanding of public health responses to potential future disease outbreaks (e.g., new variants of COVID-19 or future epidemics/pandemics). The pandemic imposed short-term and long-term challenges on the food retail industry, resulting in a significant shift in consumer indoor shopping behavior^34^. To reduce the spread of the virus, many stores implemented risk-mitigating policies to create a safer shopping environment for their customers. Since a satisfactory shopping experience is crucial to maintaining long-term profitability in the contemporary retailing industry^35^, it is important to consider public opinions toward these store policies. Understanding these opinions helps cultivate an environment that matches consumer preferences and needs, while ensuring proper measures are taken to promote public safety^25,36,37^. However, research on individual perspectives on risk-mitigation policies in food retail stores is limited. This study helps improve the understanding of public opinions regarding COVID-19-related policies in US food retail stores.

Forth, our study contributes to the literature investigating the level of public trust in governments and public health officials during the pandemic. Higher level of confidence in the government can enhance individuals’ resilience to adapt and recover from epidemics and pandemics^38^. Efficient government communication is challenging, yet essential for raising awareness and promoting risk-mitigating behaviors during global health crises. Alders and Bagnol^58^ showed that effective communication was a crucial element in the prevention and control strategy targeted toward the Highly Pathogenic Avian Influenza (HPAI), which is another deadly and highly infectious respiratory virus commonly referred to as the “bird flu”. It is therefore crucial to understand the level of public trust in these entities to increase compliance levels and enhance communication strategies. However, it is not surprising to expect individuals to have divergent attitudes and levels of trust toward different groups/organizations—such as the World Health Organization (WHO), Centers for Disease Control and Prevention (CDC), and US government—especially during global pandemics like COVID-19. Two separate studies by Soares et al.^27^ and Latkin et al.^7^ found that individuals with stronger opposition to the vaccine generally hold more negative perceptions and lower levels of trust in the CDC. Our study provides insights regarding the disparate public perspectives on the COVID-19-related performance of different organizations/groups to help governments achieve efficient communications with the public.

## Materials and methods

An online survey was conducted between June 25 and August 24, 2021, to collect data from a nationally representative sample of 1050 US adults (ages 18 and above). Proportional representation was achieved based on age, gender, income, and region (Northeast, Midwest, West, South). To ensure data quality, attention check questions were included in the questionnaire to filter out subjects who were not paying close attention to the questions^39^. In addition, the survey was limited to 15–20 min in length to avoid respondent fatigue. A copy of the survey instrument is provided in the appendix.

### Vaccination behaviors and reasons

The survey collected information related to the respondents’ vaccination behaviors. Respondents reported whether they had received at least one dose of any COVID-19 vaccine (i.e., Pfizer, Moderna, Johnson and Johnson, etc.). For unvaccinated respondents, follow-up questions were asked about future vaccination plans (i.e., whether or not they plan to get vaccinated at some point or if they are unsure). We used these responses as one of our primary outcome variables to investigate how behavioral and sociodemographic characteristics correlate with people’s vaccination decisions.

To better understand people’s vaccination hesitancy, those who indicated an unwillingness to get vaccinated were asked to specify the primary reasons for this decision, and the importance (on a scale of 0–100) of each reason, out of a total of 20 possible reasons, which included *“I am concerned about negative side effects of the vaccine”*, *“I don’t believe the vaccine is safe”*, *“I don’t believe the vaccine is effective”*, *“I don’t trust vaccines in general”*, and *“I am taking enough precautionary measures to avoid contracting COVID-19”*. The full list of vaccination-discouraging reasons used is included in Fig. 3, which is discussed in the Results section.

Respondents were also asked to report changes in their participation in 15 daily activities post-vaccination (or expected changes for unvaccinated respondents). The activities included indoor dining, outdoor dining, restaurant food pickup, restaurant food delivery, going out to bars, grocery store visits, online shopping, movie theatres, sports games, theme parks, outdoor recreation, and air travel. The respondents’ reported changes in participation in these 15 activities were then analyzed to determine factors affecting post-vaccination (anticipated) behaviors.

### Public attitudes toward store policies and levels of trust in organizations

To better understand people’s views about retail store policies and trust in different information sources, we asked respondents to indicate their level of agreement, on a Likert scale from 1 (“strongly disagree”) to 5 (“strongly agree”), with a set of 10 different grocery store policies related to COVID-19. These statements included *“grocery stores should follow CDC guidelines”*, *“the store should wipe down shopping carts”*, *“shoppers should maintain 6 feet of distance”*, *“physical barriers should be used between cashiers and customers”*, and *“masks should be worn by all customers”*. The full list is included in Fig. 5 and discussed in the Results section. Further, the survey collected respondents’ perspectives on the pandemic-related performance of different organizations—namely the WHO, foreign governments, the US government, the CDC, state health departments, local county health departments, primary care physicians, and pharmacists. Respondents indicated their evaluation of how well each entity handled the COVID-19 pandemic on a scale ranging from 1 (“not well at all”) to 5 (“extremely well”).

### Risk measurements

Four risk measures were used to elicit respondents’ risk preferences and risk perceptions, which serve as explanatory variables in our analysis. Individual risk perceptions can be measured by collecting respondents’ views of the risk involved in different risky activities^40^. Based on an approach by Lusk and Coble^56^, we measured risk perceptions related to the pandemic by collecting respondents’ perceptions of the likelihood of exposure to COVID-19 in 14 different locations/activities: grocery store, hospital/clinic, doctor’s office, delivered packages, restaurant/bar, gas station, work, home, outdoor recreation, religious gathering, public transit, taxi/ride-share, daycare, and school. Respondents reported their perceived likelihood of contracting COVID-19 in each location/activity on a Likert scale ranging from 1 (“extremely unlikely”) to 5 (“extremely likely”). We then summed the number of places where each respondent perceived the likelihood of exposure to COVID-19 as *“somewhat likely”* or “*extremely likely*” to construct the Number of Risky Places (NRP) as our first risk measure.

A more direct way of eliciting individuals’ risk perceptions related to COVID-19 is to ask respondents about their perceived probability of contracting the virus. Respondents reported on a scale from 0 to 100% their perceived likelihood that they, or someone in their household, would contract COVID-19 in 2021, which we used as the second risk measure. This measure was labeled Perceived Probability of Contracting COVID-19 (PPCOV). This risk measure is crucial given the rapidly evolving nature of the disease, the repeated COVID-19 infections among some individuals, and the assumption that respondents’ risk-related behaviors are more likely to be driven by their own subjective perceptions rather than the objective probability of contracting COVID-19.

Following the 11-point Likert scale used in the German Socio-Economic Panel (SOEP) survey^41^, a proper way to measure risk preferences is to ask participants to assess and rank their own behaviors on a continuum ranging from risk-averse to risk-loving. This approach has also been used to elicit individual risk preferences in health-related decision-making^42^. We used a generic 11-point Likert scale, ranging from 0 (“extremely risk averse”) to 10 (“extremely risk loving”), and asked respondents to identify themselves on this scale. This was taken as our third risk measure, labeled Self-Assessed Risk Aversion (SARA). This risk measure is considered adequate since it is easy for subjects to understand and follow. Also, its domain-general nature addresses the fact that the impacts of COVID-19 do not easily fit into a single domain-specific category.

The fourth risk measure used in this study elicited the number of COVID-19 mitigation behaviors, out of a total of 7, that respondents were practicing. These behaviors included maintaining physical distancing, reducing non-essential trips away from home, washing hands more frequently, wearing a mask when away from home, wearing gloves when away from home, using delivery services, and additional household cleaning/sanitation. The total number of mitigation behaviors was summed for each respondent to create the fourth risk measure, which is labeled sum of mitigation behaviors (SMB).

### COVID-19-related household information

In addition to the respondents’ sociodemographic characteristics, the survey collected COVID-19-related household information, such as the presence of immunocompromised family members and the presence of family members working in healthcare and other essential industries. Information about the respondents’ exposure to COVID-19 was also collected, namely, whether they tested positive for the virus, self-isolated, or were hospitalized due to COVID-19. The survey also elicited respondents’ outlook on COVID-19 and the economy by asking the dates (month and year) when they believed the pandemic would end and the economy would return to normal. The description of all the variables collected in our study are summarized in Table 1.

**Table 1:** Variable description.

Variable	Description
Outcome of interest	
Vaccination behavior	0 = unvaccinated; 1 = vaccinated
Vaccination intention	0 = unvaccinated and not planning to get vaccinated; 1 = unvaccinated and unsure/maybe; 2 = unvaccinated but planning to get vaccinated
Risk measures	
SMB	0–7; sum of mitigation behaviors
NRP	0–14; number of risky places
SARA	0–11; self-assessed risk aversion
PPCOV	0–100; perceived probability of contracting COVID-19
COVID-19 related variables	
Months willing to maintain These mitigation behaviors	Discrete; ranging from 1 to 13
Self-isolated	1 = yes; 0 otherwise
Tested positive	1 = yes; 0 otherwise
Hospitalized	1 = yes; 0 otherwise
Optimism of COVID-19 end	In how many months they think COVID-19 will end
Optimism of economy return	In how many months they think the economy will return to normal
Household member in essential industry	1 = yes; 0 otherwise
Household member in healthcare industry	1 = yes; 0 otherwise
Household member immunocompromised	1 = yes; 0 otherwise
Household member pregnant	1 = yes; 0 otherwise
Household member children	1 = yes; 0 otherwise
Household member senior	1 = yes; 0 otherwise
Number of family members	Discrete value
Income decreased during COVID-19	1 = yes; 0 otherwise
Socio-demographic variables	
Married	1 = yes; 0 otherwise
Republican	1 = republican; 0 otherwise
Female	1 = yes; 0 otherwise
Age	Continuous
Education	1 = less than high school degree; 2 = high school; 3 = some college but no degree; 4 = associate degree; 5 = bachelor’s degree; 6 = graduate or professional degree
Employed	1 = yes; 0 otherwise
Income	1 = under $10,000; 2 = $10,000 to $14,999; 3 = $15,000–24,999; 4 = $25,000–$34,999; 5 = $35,000–$49,999; 6 = $50,000–$74,999; 7 = $75,000–$99,999; 8 = $100,000–$149,999; 9 = $150,000–$199,999; 10 = $200,000 or more
Caucasian	1 = yes; 0 otherwise
African American	1 = yes; 0 otherwise
Asian	1 = yes; 0 otherwise
Hispanic	1 = yes; 0 otherwise

### Ethical approval

This study was approved by the Institutional Review Board of the University of Florida. Informed consent was obtained from all subjects who participated in this study. All methods were carried out in accordance with relevant guidelines and regulations.

## Results

### Descriptive statistics

The demographic and behavioral characteristics of the respondents are summarized in Table 2. Approximately, 51% of respondents are male, and the average household size pre-COVID-19 was between 2 and 3 members, with approximately 32% having children under 18 years old and 36% having senior family members. About 27% of the respondents self-identified as Republican, 39% as Democrats, and the rest as independent/other.

**Table 2:** Summary of demographic and behavioral characteristics.

Variable	%	Variable	%
Male	51	1 = Less than $10,000	7
Female	49	2 = $10,000–$14,999	4
		3 = $15,000–$24,999	7
1 = 18–24	8	4 = $25,000–$34,999	12
2 = 25–34	14	5 = $35,000–$49,999	12
3 = 35–44	18	6 = $50,000–$74,999	18
4 = 45–54	16	7 = $75,000–$99,999	13
5 = 55–64	18	8 = $100,000–$149,999	18
6 = 65 or more	26	9 = $150,000–$199,999	6
		10 = $200,000 or more	4
Under 5-years-old	12		
6–12 years old	20	Income Decrease due to COVID-19	27
13–17 years old	18		
Under 18 (all categories)	32	Republican	27
		Democrat	39
65 years or older	36	Other	34
1 = Less than high school	5	Household Member Immunocompromised	18
2 = High school/GED	21	Household Member Pregnant	4
3 = Some college	20	Household Member Essential Worker	37
4 = Associates degree	14		
5 = Bachelor’s degree	23	Vaccinated	72
6 = Grad./professional degree	17	Unvaccinated, Planning to	7
		Unvaccinated, Unsure/Maybe	8
		Unvaccinated, Not Planning to	13

	Mean	Median	Std. Dev
Household size prior to COVID-19	2.75	2	1.65
Self-assessed risk aversion (SARA)	4.73	5	2.72
Number of risky places (NRP)	3.87	4	4.22
Perceived probability of contracting COVID-19 (PPCOV)	21.2	10	23.77
Sum of mitigation behaviors (SMB)	3.97	4	1.80

Approximately 72% of the respondents claimed that they or their spouse had received at least one dose of any brand of the COVID-19 vaccine. Among unvaccinated respondents, 25% (7% of total sample) reported an intention to get vaccinated in the future, 29% (8% of total sample) were unsure whether or not they would want to receive the vaccine, and the remaining 46% of the unvaccinated respondents (13% of total sample) indicated a preference against being vaccinated. Regarding other health-related variables, 18% of the respondents reported having immunocompromised members in their household, while only 4% had pregnant household members. About one-third of the respondents reported household members employed in essential industries, and 27% reported a reduction in their household income level due to COVID-19.

With regard to the risk measures, we find that the average SARA was 4.73 out of 10, which indicates slight risk aversion. On the other hand, the mean NRP was only 3.87, implying that respondents only perceived a positive risk in an average of 4 out of 14 places/activities. The average PPCOV for the sample was approximately 21%. A correlation matrix is included in the online appendix to examine potential multicollinearity issues between our risk measures. Based on the calculated Variance Inflation Factors (VIFs), which were all quite low, we find no evidence of significant multicollinearity between any of our measures.

**Figure 1:**
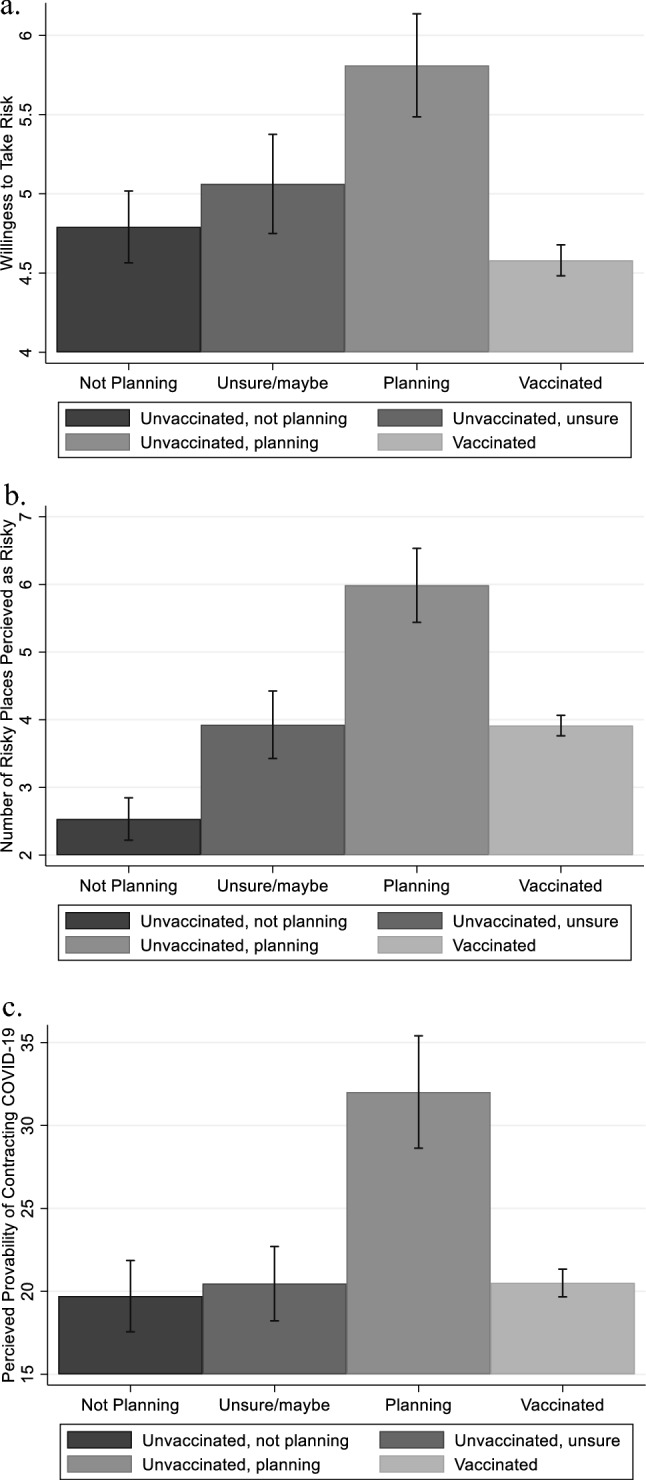
Histograms for Risk Measures. (**a**) Self-Assessed Risk Aversion (SARA); (**b**) Number of Risky Places (NRP); (3) Perceived Probability of Contracting COVID-19 (PPCOV).

Since these risk measures would most likely differ with respondents’ vaccination decisions and plans, bar graphs of SARA, NRP, and PPCOV of respondents with different vaccination decisions/plans are shown in Fig. 1. Among all the unvaccinated respondents, those who reported an intention to get vaccinated in the future have the highest mean value of SARA (5.81), indicating that they were more willing to take risks than individuals who do not intend to get vaccinated or who are unsure. This might imply that a majority of unvaccinated respondents view getting vaccinated as a risky behavior, which could explain the lack of intent to get vaccinated in order to avoid potential risks associated with the vaccine. Vaccinated respondents, on the other hand, were the most risk-averse group among all. This may be caused by the fact that they perceived COVID-19 as riskier than the vaccine. The group of respondents who were unvaccinated and not planning to get vaccinated had a mean NRP value of 2.53 and thus perceived the least number of places as risky. Those who were unvaccinated but planning to get vaccinated perceived the highest number of risky places, with a mean NRP of 5.98. In terms of PPCOV, unvaccinated individuals who had the intention to receive the vaccine in the future reported the highest level of PPCOV (32%). The average PPCOV of vaccinated individuals was 21%. It is highly probable that these individuals believe that getting vaccinated was effective, and it significantly decreased their perceived probability of contracting COVID-19. Unvaccinated individuals who did not intend to receive the vaccine had the lowest level of PPCOV (19.71) among all groups of respondents.

**Figure 2:**
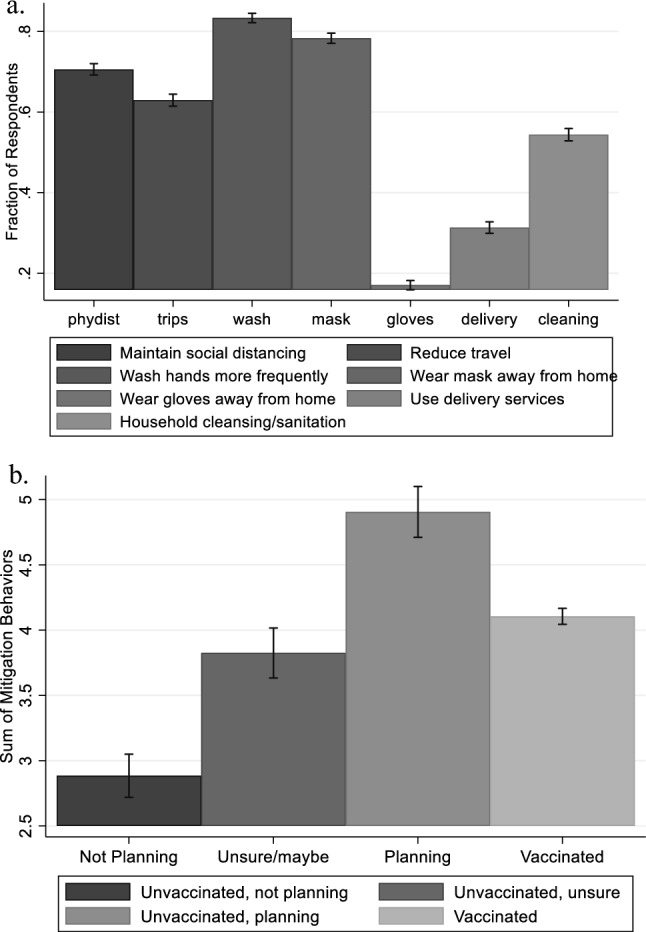
Participation in Risk Mitigation Behaviors. (**a**) Fraction participating in each risk mitigation behavior; (**b**) histogram for Sum of Mitigation Behaviors (SMB).

Figure 2 presents the fraction of respondents following each of seven COVID-19 risk-mitigation behaviors, along with a breakdown of SMB by respondents’ vaccination decisions or plans. The mean value of SMB was 3.97 out of 7. We observe that most people were practicing frequent hand washing (83%), wearing a mask away from home (78%), and maintaining social distancing (70%). Conversely, the least practiced risk-mitigation behavior related to COVID-19 was “wearing gloves away from home”. Unvaccinated individuals who reported an intention to get vaccinated in the future had the highest value of SMB (4.91) among all groups of respondents, followed by the vaccinated group (4.11). Individuals who were unvaccinated and did not plan to get vaccinated reported practicing the fewest risk-mitigating behaviors (2.89).

### Analyzing vaccination behaviors

**Figure 3:**
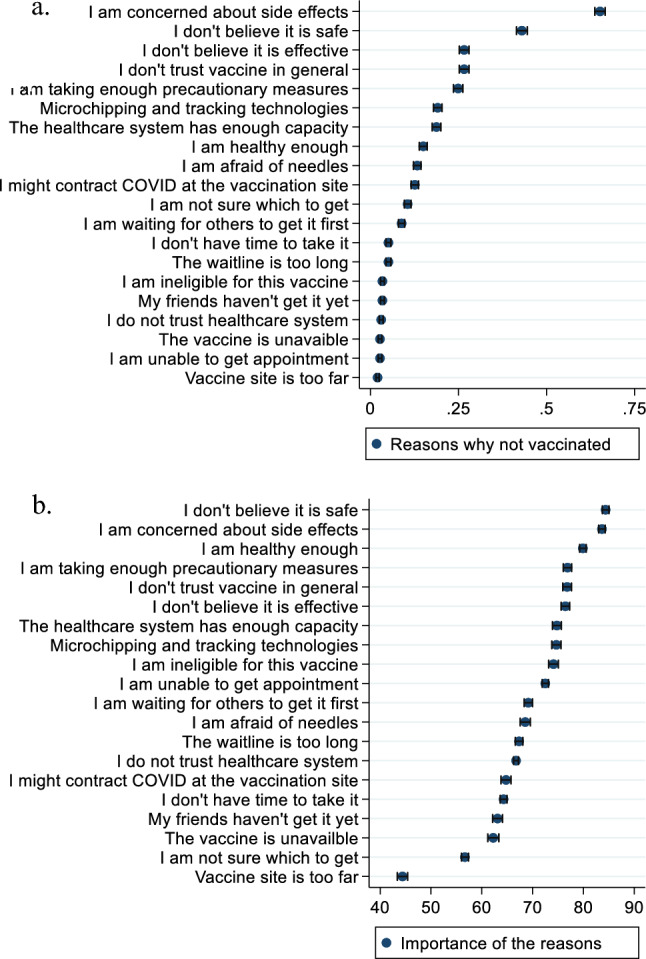
Reasons for not receiving COVID-19 vaccination. (**a**) fraction of respondents selecting each reason. (**b**) average importance of each reason for respondents.

Figure 3 presents a summary of the reasons why respondents choose not to receive COVID-19 vaccines, as well as the perceived importance of each reason. The most common reason was concern about the potential side effects of the COVID-19 vaccines, which was selected by approximately 65% of respondents. This was followed by the safety concern, where 43% reported that they do not believe the vaccine is safe. Approximately 26% of the unvaccinated group doubted the effectiveness of the COVID-19 vaccine, another 26% did not trust any vaccine in general, and about 25% believed they were taking enough precautionary measures. The least chosen reasons for not receiving the COVID-19 vaccines were “the vaccine is unavailable,” “I am unable to get an appointment,” and “the vaccine site is too far” (Fig. 3a). With an average perceived importance score of 84 out of 100, concern about the safety was perceived as the most important reason for not receiving the COVID-19 vaccines (Fig. 3b). The second and third most important reasons were concerns about side effects and confidence in one’s health condition, with scores of 83 and 79, respectively. Reasons related to the availability and convenience of obtaining the COVID-19 vaccines received the lowest importance ratings by the respondents.

Two separate regressions were used to investigate factors associated with vaccination decisions. The first regression was a Logistic model over the full sample, with an indicator outcome variable that took the value 1 for vaccinated and 0 for unvaccinated. The second regression was estimated using the unvaccinated subsample, where we coded unvaccinated respondents with a 3-level outcome variable that took the value 0 if the respondent had no intention of being vaccinated, 1 if they were unsure, and 2 if they planned to be vaccinated in the future. Since the outcome variable is ordered in an increasing tendency toward vaccination, we estimated an ordered Logit model over the unvaccinated subgroup. This allows us to assess how different factors correlate with both current and future vaccination plans.

Table 3 presents the results of both regression models.We also use the unvaccinated subsample to estimate a multinomial logit model. The results are presented in Appendix A. In the logistic model, we observe that individuals who follow more COVID-19-related risk mitigation behaviors are more likely to be vaccinated. Not surprisingly, SARA was negatively correlated with people’s vaccination decisions, indicating that a more risk-averse person (i.e., lower SARA) was more likely to get the vaccine. PPCOV was also negatively associated with respondents’ vaccination decisions, which implies a higher likelihood of being vaccinated for individuals with a lower perceived probability of contracting COVID-19. Having self-isolated also increased the likelihood of getting the vaccine, while being optimistic about economic recovery post-COVID-19 had an opposite effect.

**Table 3:** Factors correlated with the likelihood of accepting the vaccine and plans.

Variable	Vaccination behavior	Vaccination plan for unvaccinated group
	Logit odds ratios	Ordered logit odds ratios
Sum of mitigation behaviors (SMB)	0.127***	0.341***
	(0.049)	(0.079)
Number of risky places (NRP)	0.023	0.105***
	(0.023)	(0.035)
Self-assessed risk aversion (SARA)	− 0.101***	0.010
	(0.032)	(0.050)
Perceived probability of contracting COVID-19 (PPCOV)	− 0.008**	0.007
	(0.004)	(0.006)
Months willing to maintain these mitigation behaviors	− 0.017	− 0.021
	(0.019)	(0.031)
Self-isolated	0.543***	0.215
	(0.177)	(0.313)
Tested positive	− 0.398*	0.268
	(0.219)	(0.369)
Hospitalized	− 0.270	0.170
	(0.215)	(0.371)
Optimism of COVID-19 end	0.002	− 0.009
	(0.004)	(0.006)
Optimism of economy return	− 0.010***	− 0.003
	(0.003)	(0.006)
Household member in essential industry	0.139	− 0.257
	(0.194)	(0.309)
Household member in healthcare industry	− 0.077	0.479
	(0.248)	(0.412)
Household member immunocompromised	0.550**	− 0.635
	(0.230)	(0.402)
Household member pregnant	0.132	0.554
	(0.423)	(0.740)
Household member children	− 0.682***	0.596*
	(0.227)	(0.334)
Household member senior	0.489**	− 0.814**
	(0.213)	(0.355)
Number of family members	− 0.044	0.026
	(0.060)	(0.096)
Income decreased	0.045	− 0.402
	(0.183)	(0.311)
Marriage	0.419**	0.020
	(0.179)	(0.289)
Republican	− 0.734***	− 0.731**
	(0.178)	(0.300)
Female	− 0.167	− 0.241
	(0.165)	(0.278)
Age	0.153**	0.269**
	(0.068)	(0.113)
Education level	0.199***	− 0.201*
	(0.061)	(0.106)
Employed	− 0.072	− 0.189
	(0.215)	(0.315)
Income level	0.078*	0.160**
	(0.043)	(0.070)
White	0.974**	− 0.478
	(0.383)	(0.616)
Black	− 0.019	− 0.675
	(0.422)	(0.678)
Asian	2.604***	15.066
	(0.723)	(904.650)
Hispanic	0.841***	0.744
	(0.307)	(0.523)
/cut1		1.570
		(0.972)
/cut2		3.221***
		(0.989)
Constant	− 1.118*	
	(0.653)	
Log likelihood	− 513.133	− 248.504
Observations	1,047	292

Standard errors in parentheses. ***p < 0.01, **p < 0.05, *p < 0.1; A full list of description of variables are included in Table 1.

Regarding other highly significant demographic variables, having children in the household decreased the likelihood of getting the vaccine, whereas the presence of senior or immunocompromised household members increased the probability of getting the vaccine. We also find lower vaccination likelihood among respondents identifying as Republicans compared to other political groups. On the other hand, respondents who were married, older, and/or more educated had a significantly higher likelihood of being vaccinated. Compared to other ethnicities, results also imply that being either White, Asian, or Hispanic American significantly increased the likelihood of receiving the COVID-19 vaccine.

With respect to the ordered logit model over the unvaccinated subsample, our results consistently show a positive correlation with SMB, indicating that, among unvaccinated individuals, those practicing more mitigation behaviors have higher intentions to get vaccinated in the future. NRP was also highly associated with respondents’ vaccination plans, suggesting that respondents identifying a positive risk of COVID-19 infections in a larger number of places are more inclined toward planning to receive COVID-19 vaccination. Other risk measures investigated (i.e., PPCOV and SARA) were not significantly correlated with respondents’ plans to receive COVID-19 vaccination. Notably, we find a negative association between having senior household members and planning future COVID-19 vaccination among unvaccinated respondents, which is contrary to the result in the Logit model using the full sample. This implies that, although having senior household members increased the likelihood of receiving COVID-19 vaccination, individuals who were not yet vaccinated were less likely to plan to do so if they had senior members in the household. The role of political affiliation and age on vaccination plans was consistent with the sign of their correlation with vaccination decisions. Specifically, self-identified Republicans and younger individuals are less likely to plan COVID-19 vaccination. Finally, respondents with higher income levels had a higher likelihood of planning to get vaccinated. 

### Analyzing changes in activities post-vaccination

**Figure 4:**
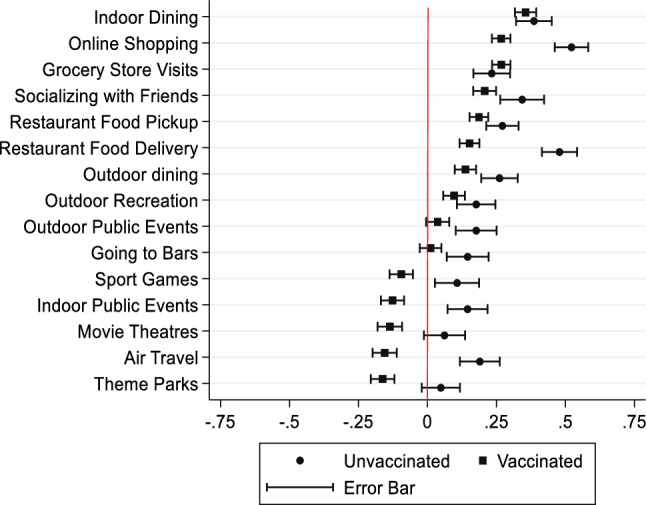
Changes in the level of participation of vaccinated and unvaccinated individuals.

Changes in the level of participation in various activities for vaccinated respondents after receiving their vaccinations, and the anticipated changes for unvaccinated individuals, are summarized in Fig. 4. Values that are greater than 0 indicate an increase in the level of participation in a particular activity, and vice versa. Unvaccinated respondents reported higher engagement in almost all categories of activities compared to vaccinated respondents. On average, vaccinated individuals reported the highest increase in indoor dining, online shopping, and grocery store visits post-vaccination. On the other hand, unvaccinated respondents anticipated the highest increase in online shopping, restaurant food delivery, indoor dining, and socializing with friends upon vaccination. Notably, vaccinated respondents reported a decrease in their participation in organized sport games, indoor public events, movie theatres, air travel, and theme parks post-vaccination. In contrast, unvaccinated respondents did not report a decrease in participation in any of the 15 activities listed. One possible explanation for this could be the fact that vaccinated individuals are the most risk-averse group among all other groups of respondents, as presented earlier in Fig. 1a.

To further investigate factors that are correlated with (anticipated) changes in participation in the 15 activities/places, we first conducted a Principal Component Analysis (PCA) as a method to reduce the dimensionality of the data while preserving the majority of observed variation^43^. Using this data reduction technique, a series of factors can be described by fewer inter-correlated quantitative variables (i.e., linear combinations of these factors of interest) to facilitate data analysis. Due to the fact that many of the 15 factors are moderately or highly correlated, we did not choose to perform a Cluster Analysis (CA) since CA will generally be outperformed by PCA in cases where the variables of interest are highly correlated^44^. To test the suitability of PCA, we performed 3 different tests, namely Kaiser–Meyer–Olkin (KMO) Test, Bartlett’s Test of Sphericity, and the Cronbach’s Alpha Test. We obtained an overall score of 0.9236 for the KMO Test, implying a high sampling adequacy level^45^. Bartlett’s Test of Sphericity also showed high significance with a *p*-value of 0.000^45^. The scale reliability coefficient obtained from Cronbach’s Alpha Test was 0.893, which indicated a high level of internal consistency in our sample data^46^. Thus, we concluded that the PCA was appropriate for our analysis.

Three principal components were found to be the best fit, explaining approximately 63.57% of the variability in the data. Factor loadings of each principal component on the activities are reported in Table 4. The first component, defined as “high social-exposure activities” (HSA), included going to bars, movie theatres, organized sports games, theme parks, air travel, indoor public events, and outdoor public events. These activities are generally perceived as highly risky since they involve interacting with a large number of individuals. Also, many of these were recreational activities that could be considered unnecessary (compared to other essential daily activities), and the pandemic was negatively impacting their corresponding industries. The second component was labeled “medium social-exposure activities” (MSA) and comprised indoor dining, outdoor dining, grocery store visits, and socializing with close friends. These activities required people to interact with relatively smaller crowds and could thus be considered intermediate in risk level. The third component, labeled “low social-exposure activities” (LSA), included restaurant food pickup, restaurant food delivery, and online shopping. This category of activities required minimum contact with other people and could thus be considered low risk.

**Table 4:** Loading factors of each principal component on all the activities/places.

Variable	High social-exposure activities	Medium social-exposure activities	Low social-exposure activities
Theme parks	0.4325		
Sports games	0.4310		
Movie theatres	0.3928		
Indoor public events	0.3593		
Air travel	0.3448		
Bars	0.2982		
Outdoor public events	0.2546		
Indoor dining		0.5441	
Outdoor dining		0.4749	
Grocery store visits		0.4337	
Socializing with close friends		0.4052	
Restaurant food delivery			0.6104
Restaurant food pickup			0.5518
Online shopping			0.5473

We used 0.25 as the threshold, and thus 14 out of 15 activities/places were able to be categorized into three principal components.

Separate regressions were estimated using the three components from PCA as outcome variables, as presented in Table 5. The same covariates from Table 3 were included in these regressions. Regression results indicated that vaccinated respondents were more prone to engage in MSA. Planning to receive the vaccine in the future resulted in a higher level of participation in LSA. We also find that respondents following a higher number of COVID-19 risk-mitigation behaviors decrease their participation in HSA and MSA activities. Furthermore, results suggested a highly positive correlation between NRP and engagement in activities from all three categories. The positive coefficients on SARA implied that risk-loving respondents tend to participate more in both HSA and LSA. We also observed that a higher PPCOV was associated with an increase in participation in LSA.

**Table 5:** Factors correlated with social-exposure activities of three levels.

Variable	High social-exposure activities	Medium social-exposure activities	Low social-exposure activities
Vaccinated	− 0.200	0.319***	0.143*
	(0.133)	(0.108)	(0.083)
Planning vaccination	0.076	0.325*	0.269**
	(0.218)	(0.177)	(0.136)
Sum of mitigation behaviors (SMB)	− 0.085***	− 0.057**	0.029
	(0.031)	(0.025)	(0.019)
Number of risky places (NRP)	0.040***	0.060***	0.046***
	(0.014)	(0.011)	(0.009)
Self-assessed risk aversion (SARA)	0.041**	0.027*	0.036***
	(0.020)	(0.016)	(0.012)
Perceived probability of contracting COVID-19 (PPCOV)	0.003	0.001	0.003**
	(0.002)	(0.002)	(0.001)
Months willing to maintain mitigation behaviors	0.006	− 0.006	− 0.007
	(0.012)	(0.010)	(0.007)
Self-isolated	0.229**	0.107	0.036
	(0.107)	(0.087)	(0.067)
Tested positive	− 0.027	− 0.064	0.174**
	(0.133)	(0.108)	(0.083)
Hospitalized	− 0.123	− 0.150	− 0.011
	(0.128)	(0.104)	(0.080)
Optimism of COVID-19 end	− 0.003	− 0.002	0.002
	(0.002)	(0.002)	(0.002)
Optimism of economy recovery	− 0.003	− 0.001	− 0.004***
	(0.002)	(0.002)	(0.001)
Household member in essential industry	0.011	− 0.013	− 0.045
	(0.122)	(0.099)	(0.076)
Household member in healthcare industry	0.099	− 0.086	− 0.209**
	(0.154)	(0.125)	(0.096)
Household member immunocompromised	0.107	− 0.002	0.116
	(0.133)	(0.108)	(0.083)
Household member pregnant	0.502*	0.365*	0.444***
	(0.262)	(0.213)	(0.164)
Household member children	− 0.046	0.000	0.239***
	(0.145)	(0.118)	(0.091)
Household member senior	0.024	− 0.110	− 0.167**
	(0.132)	(0.107)	(0.083)
Number of family members	0.017	0.015	− 0.020
	(0.039)	(0.032)	(0.025)
Income decreased	− 0.066	− 0.032	− 0.065
	(0.114)	(0.092)	(0.071)
Marriage	0.039	0.032	0.119*
	(0.112)	(0.091)	(0.070)
Political affiliation	− 0.018	− 0.021	− 0.071
	(0.114)	(0.092)	(0.071)
Female	0.025	0.107	− 0.053
	(0.102)	(0.083)	(0.064)
Age	− 0.026	0.010	− 0.012
	(0.044)	(0.036)	(0.027)
Education level	0.048	0.042	0.055**
	(0.038)	(0.031)	(0.024)
Employed	0.093	0.107	− 0.151*
	(0.133)	(0.108)	(0.083)
Income level	0.001	0.045**	0.017
	(0.028)	(0.022)	(0.017)
White	− 0.057	0.001	0.074
	(0.239)	(0.194)	(0.150)
Black	− 0.193	− 0.120	0.131
	(0.263)	(0.213)	(0.165)
Asian	− 0.099	0.047	0.013
	(0.323)	(0.262)	(0.202)
Hispanic	− 0.442**	− 0.451***	− 0.028
	(0.184)	(0.149)	(0.115)
Constant	− 0.117	− 0.461	− 0.552**
	(0.411)	(0.334)	(0.257)
Observations	1,047	1,047	1,047
R-squared	0.071	0.116	0.194

Standard errors in parentheses. ***p < 0.01, **p < 0.05, *p < 0.1; A full list of description of variables are included in Table 1.

Regarding other control variables, having self-isolated increased respondents’ participation in HSA post-vaccination. However, participants who tested positive were more prone to engage in LSA. Unsurprisingly, results also suggested that respondents who are more optimistic about economic recovery increased their participation in LSA. In addition, having pregnant members or children in the household increased engagement in LSA, while the presence of seniors or members in the healthcare industry decreased engagement in LSA. Results also suggest that respondents with higher education levels were more likely to participate in LSA, while higher income respondents were more likely to engage in MSA. Finally, Hispanic participants were less likely to engage in both HSA and MSA.

### Analyzing public opinions towards grocery store policies and public health safety efforts of different organizations

**Figure 5:**
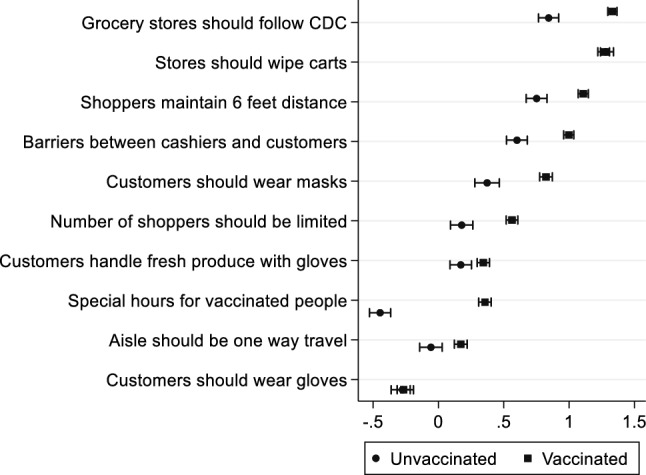
Level of agreement on store policies.

Given the respondents’ reported increase in grocery store visits, and their participation in risk-mitigation behaviors, it is crucial to understand their opinions on various store policies to mitigate the transmission of COVID-19 infections. The participants’ average agreement with various grocery store policies was coded in a 5-point Likert scale, ranging from − 2 (strongly disagree) to 2 (strongly agree). Results are presented in Fig. 5. Notably, the vaccinated group reported higher agreement with all store policies compared to unvaccinated respondents. The highest level of agreement was given to “Grocery stores should follow CDC recommendations”, “The store should wipe shopping carts”, and “Shoppers should maintain 6 feet of distance”. While most store policies received positive feedback from the respondents, both the vaccinated and unvaccinated groups opposed the requirement of mandatory glove-wearing by all customers. Further, the unvaccinated group disagreed that “There should be special access hours for people who are vaccinated” and that “Aisles should be one-way travel”. These results further confirm that vaccination does not make respondents open to more risky activities. Instead, it is highly probably that risk preference is what drives individuals’ risk behaviors and attitudes toward grocery store policies.

**Figure 6:**
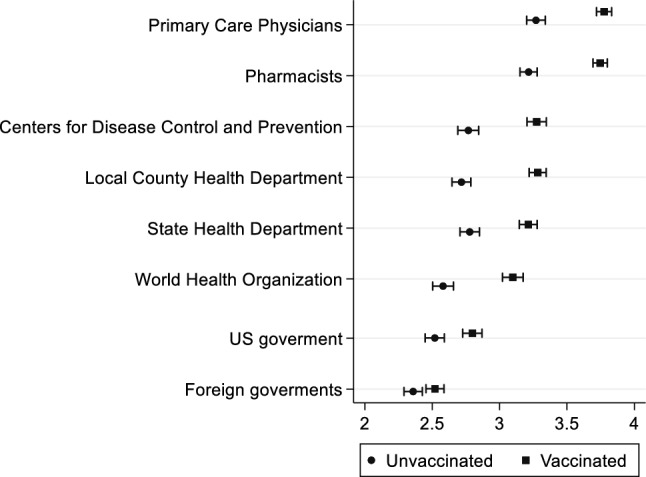
Rating how well each group handled public health safety efforts related to COVID-19.

Respondents’ perspectives on how well different organizations handled public health safety efforts related to COVID-19 were coded in a 5-point Likert scale ranging from 1 (not well at all) to 5 (extremely well). Results are summarized in Fig. 6. We find that vaccinated individuals gave a higher assessment of all the groups/organizations, on average, compared to unvaccinated respondents. Among all the organizations, primary care physicians and pharmacists were highly recognized by both vaccinated and unvaccinated respondents for their efforts related to the COVID-19 pandemic. Further, both groups of respondents gave the lowest ratings to the US and Foreign governments.

## Discussion and conclusion

The COVID-19 pandemic had a negative impact on the world, not only because of COVID-19’s high transmission, infection, and mortality rates when compared to previous respiratory diseases, but also because of the social consequences, such as economic loss due to limited operations or even closure of many businesses, involuntary unemployment, and high public health expenses^47–49^. Thus, it is crucial to investigate individuals’ vaccine-related behaviors to provide useful insights for public health policies to achieve higher compliance and further promote vaccine confidence among the US population to establish herd immunity and prevent further economic losses.

In this study, we investigated primary factors influencing people’s vaccination decisions to better understand their vaccine hesitancy. We customized four different risk measures relating to both risk perceptions and risk preferences and analyzed their relative correlation with respondents’ vaccination decisions. Results indicate that all four risk measures were highly associated with respondents’ vaccination behaviors, and vaccination plans for unvaccinated individuals. In addition to the risk measures, our study conforms to previous findings that reported significant associations between sociodemographic variables, such as race, age, political affiliation, education, and income level, and individuals’ vaccination behaviors^15,16,27,50,51^. However, unlike previous studies by Akarsu et al.^13^, Troiano and Nardi^51^, and Soares et al.^27^, our results suggest that gender, working status, and experiencing income loss due to the pandemic do not significantly influence the decision to get vaccinated.

When looking at the main reasons why unvaccinated individuals are reluctant to get vaccinated, we discover that a majority were highly concerned about side effects, effectiveness, and safety of the vaccine, which is consistent with previous findings^13,18,19,26,27^. Some respondents were overly optimistic about their personal health conditions or mistrusted vaccines in general. More importantly, our results suggest that for a vast majority of individuals, it is not convenience or availability that is stopping them from getting vaccinated. Instead, anti-vaccine sentiments seem to be the most crucial reason. This implies that rather than putting more effort into increasing the availability of the vaccine, efforts should be directed at informational and promotional campaigns to encourage higher vaccine trust and acceptance. These results can help guide policymakers in promoting higher vaccine uptake, not only specific to COVID-19, but also for the vaccines created for other infectious diseases. Specifically, our results highlight the importance of assuring vaccine effectiveness and addressing public concerns over vaccine safety and potential side effects. In addition, policymakers can use these findings to tailor their promotional campaigns to specific groups of people who were shown to carry stronger anti-vaccine sentiments.

The COVID-19 pandemic reshaped people’s lifestyles and their level of participation in several daily activities. The significant correlations we highlight between our risk measures and three groups of activities with different levels of social exposure provide useful implications for understanding people’s behavioral changes post-vaccination. All of the four risk measures were strongly associated with at least one of the social-exposure activities. In addition, vaccinated respondents were more prone to participate in MSA. Self-isolating or testing positive for COVID-19, optimism about economic recovery, presence of special household members (i.e., pregnant, immunocompromised, etc.), education level, and ethnicity were all significant determinants of respondents’ level of participation in different activities post-vaccination. This information is useful in guiding policymakers to effectively focus efforts on regulating high social exposure activities among groups with a higher participation likelihood to prevent further outbreaks of infections in the future. Our results also suggested that policymakers and state health departments can perform preventive health regulation by focusing on the activities/locations with high participation levels, not specific to COVID-19, but also in any future events of pandemics. These could include limiting the total number of people in a restaurant or grocery store, mandating mask wearing, and requiring negative test results, which were implemented in a majority of states/territories during the early months of the pandemic^52–55^.

Lastly, this study expands on the literature on public opinions by investigating the level of agreement with several grocery store policies, as well as levels of trust in different groups/organizations. Since both reducing the spread of the virus and ensuring customer satisfaction are highly important, the public’s perspectives on store risk-mitigation policies can help grocery stores, and other retail industries, to adjust their procedures and services to achieve higher customer satisfaction while maintaining a safe shopping environment. Furthermore, our study shows that primary care physicians and pharmacists received the highest rating and level of trust compared to other organizations/groups. At the same time, the US and foreign governments were least appreciated in terms of their health-related efforts during the pandemic. These findings provide insights regarding the disparate public opinions to help governments achieve efficient communications with the public to better address COVID-19 and other future public health challenges.

### Supplementary Information

Supplementary Information.

## Data Availability

The datasets used and/or analyzed during the current study are available from the corresponding author upon request.
