# Supplementary material for: Understanding the role of risk preferences and perceptions in vaccination decisions and post-vaccination behaviors among U.S. households
Source: Sci Rep. 2024 Feb 7;14:3190. doi: 10.1038/s41598-024-52408-6 (PMC10850518; doi:10.1038/s41598-024-52408-6)
Supplement: Supplementary file 1 — Supplementary Information. [file 41598_2024_52408_MOESM1_ESM.docx]

**Appendix. A**

**Table A1.** Correlation matrix of risk measures

| VIF | Variable | Vaccination Status | Sum of Mitigation Behaviors (SMB) | Number of Risky Places (NRP) | Self-Assessed Risk Aversion (SARA) | Perceived Probability of Contracting COVID-19 (PPCOV) |
| --- | --- | --- | --- | --- | --- | --- |
| —— | Vaccination Status | 1.0000 |  |  |  |  |
| (1.33) | Sum of Mitigation Behaviors (SMB) | 0.1131* | 1.0000 |  |  |  |
| (1.55) | Number of Risky Places (NRP) | 0.0136 | 0.3382* | 1.0000 |  |  |
| (1.26) | Self-Assessed Risk Aversion (SARA) | -0.0896* | 0.0312 | 0.2364* | 1.0000 |  |
| (1.23) | Perceived Probability of Contracting COVID-19 (PPCOV) | -0.0476 | 0.1593* | 0.3507* | 0.1169* | 1.0000 |

*Notes:* Standard errors in parentheses. * p<0.05; a full list of description of variables are included in Table 1.

**Table A2.** Factors correlated with the likelihood of planning to receive the vaccine

| Variable | Vaccination Plan for Unvaccinated Group | | | |
| --- | --- | --- | --- | --- |
|  | Not Planning | Unsure/Maybe | Planning | |
|  | Base | Multinomial  Odds Ratios | | |
| Sum of Mitigation Behaviors (SMB) |  | 0.301*** | | 0.430*** |
|  |  | (0.102) | | (0.120) |
| Number of Risky Places (NRP) |  | 0.129*** | | 0.130** |
|  |  | (0.048) | | (0.054) |
| Self-Assessed Risk Aversion (SARA) |  | 0.048 | | -0.014 |
|  |  | (0.065) | | (0.077) |
| Perceived Probability of Contracting COVID-19 (PPCOV) |  | -0.003 | | 0.015* |
|  |  | (0.007) | | (0.008) |
| Months Willing to Maintain These Mitigation Behaviors |  | -0.053 | | 0.016 |
|  |  | (0.039) | | (0.049) |
| Self-isolated |  | -0.629 | | 0.572 |
|  |  | (0.422) | | (0.457) |
| Tested Positive |  | -0.562 | | 1.137* |
|  |  | (0.459) | | (0.640) |
| Hospitalized |  | -0.333 | | 0.712 |
|  |  | (0.454) | | (0.621) |
| Optimism of COVID-19 End |  | -0.007 | | -0.013 |
|  |  | (0.008) | | (0.010) |
| Optimism of Economy Return |  | -0.003 | | -0.004 |
|  |  | (0.007) | | (0.009) |
| Household Member in Essential Industry |  | 0.341 | | -0.604 |
|  |  | (0.401) | | (0.486) |
| Household Member in Healthcare Industry |  | -0.257 | | 1.022* |
|  |  | (0.564) | | (0.601) |
| Household Member Immunocompromised |  | -0.945* | | -1.168** |
|  |  | (0.546) | | (0.573) |
| Household Member Pregnant |  | 0.556 | | 0.987 |
|  |  | (1.014) | | (1.101) |
| Household Member Children |  | 0.519 | | 0.919* |
|  |  | (0.438) | | (0.508) |
| Household Member Senior |  | -1.043** | | -1.053* |
|  |  | (0.463) | | (0.541) |
| Number of Family Members |  | 0.103 | | 0.002 |
|  |  | (0.125) | | (0.145) |
| Income Decreased |  | -0.442 | | -0.431 |
|  |  | (0.407) | | (0.466) |
| Marriage |  | -0.513 | | 0.283 |
|  |  | (0.379) | | (0.439) |
| Republican |  | -1.006*** | | -0.767* |
|  |  | (0.386) | | (0.458) |
| Female |  | 0.596 | | -0.647 |
|  |  | (0.377) | | (0.422) |
| Age |  | 0.381*** | | 0.294* |
|  |  | (0.145) | | (0.175) |
| Education Level |  | -0.314** | | -0.240 |
|  |  | (0.137) | | (0.162) |
| Employed |  | -0.534 | | -0.228 |
|  |  | (0.408) | | (0.482) |
| Income Level |  | 0.161* | | 0.225** |
|  |  | (0.091) | | (0.104) |
| White |  | -0.909 | | -0.952 |
|  |  | (0.802) | | (0.831) |
| Black |  | -0.567 | | -1.451 |
|  |  | (0.885) | | (0.920) |
| Asian |  | -1.526 | | 14.025 |
|  |  | (1,828.599) | | (1,139.583) |
| Hispanic |  | -0.385 | | 0.755 |
|  |  | (0.760) | | (0.673) |
| Constant |  | -0.866 | | -3.675** |
|  |  | (1.231) | | (1.479) |
|  |  |  | |  |
| Observations | 292 | 292 | | 292 |

*Notes:* Standard errors in parentheses. *** p<0.01, ** p<0.05, * p<0.1; a full list of description of variables are included in Table 1

The results of Multinomial Logit model over the subsample of unvaccinated respondents are summarized in Table A2. The positive coefficients on SMB indicate that practicing more risk mitigation behaviors significantly increases intentions to receive the vaccine among unvaccinated respondents. NRP is also positively associated with vaccination plans, suggesting that respondents identifying a positive risk of COVID-19 infections in a larger number of places are more inclined toward planning to receive the vaccine in the future. Other risk measures investigated (i.e., PPCOV and SARA) were not significantly correlated with the two subcategories of individuals’ vaccination plans.

We also find a negative association between having immunocompromised household members and intention to receive vaccination in the future, which is contrary to the result in the Logit model using the full sample. This implies that, although having immunocompromised household members increased the likelihood of receiving COVID-19 vaccination, individuals who were not yet vaccinated were less likely to plan for vaccination if they had immunocompromised members in the household. Having senior household members decreased unvaccinated respondents’ likelihood planning for vaccination. Results also suggest that self-identified Republicans, as well as younger respondents, were less likely to plan for vaccination if not already vaccinated. Finally, the positive coefficient of income level suggests that respondents with higher income had a higher likelihood of planning to get vaccinated.
